# Defining Conditions for Optimal Inhibition of Food Intake in Rats by a Grape-Seed Derived Proanthocyanidin Extract

**DOI:** 10.3390/nu8100652

**Published:** 2016-10-20

**Authors:** Joan Serrano, Àngela Casanova-Martí, Mayte Blay, Ximena Terra, Anna Ardévol, Montserrat Pinent

**Affiliations:** MoBioFood Research Group, Departament de Bioquímica i Biotecnologia, Universitat Rovira i Virgili, 43007 Tarragona, Spain; joanet70@gmail.com (J.S.); angela.casanova@urv.cat (À.C.-M.); mteresa.blay@urv.cat (M.B.); ximena.terra@urv.cat (X.T.); montserrat.pinent@urv.cat (M.P.)

**Keywords:** proanthocyanidins, food intake, dose, GLP-1, hypothalamus, synchronicity

## Abstract

Food intake depends on homeostatic and non-homeostatic factors. In order to use grape seed proanthocyanidins (GSPE) as food intake limiting agents, it is important to define the key characteristics of their bioactivity within this complex function. We treated rats with acute and chronic treatments of GSPE at different doses to identify the importance of eating patterns and GSPE dose and the mechanistic aspects of GSPE. GSPE-induced food intake inhibition must be reproduced under non-stressful conditions and with a stable and synchronized feeding pattern. A minimum dose of around 350 mg GSPE/kg body weight (BW) is needed. GSPE components act by activating the Glucagon-like peptide-1 (GLP-1) receptor because their effect is blocked by Exendin 9-39. GSPE in turn acts on the hypothalamic center of food intake control probably because of increased GLP-1 production in the intestine. To conclude, GSPE inhibits food intake through GLP-1 signaling, but it needs to be dosed under optimal conditions to exert this effect.

## 1. Introduction

Food intake plays a key role in body energetics and is one of the most commonly modulated parameters used to counteract obesity related problems, despite being a complex system [1,2]. In humans, the homoeostatic control of appetite is often conceptualized through a series of physiological processes that initiate and terminate feeding (i.e., satiation), and those which suppress inter-meal hunger (i.e., satiety). Collectively, these processes have been termed the Satiety Cascade and they involve psychological and behavioral patterns, peripheral physiological and metabolic events and neural and metabolic interactions in the brain [3,4,5,6]. This complex interplay of integrative neural processes within the brain, which are sensitive to a plethora of signals that originate in the periphery, underpin the behavioral expression and conscious experience of appetite and represent the ultimate factors in the control of energy intake [2].

The macronutrient composition of food play a role in the expression of the satiety cascade and appetite related processes [1]. When expressed relative to energy content rather than weight of food, protein exerts the strongest effect on satiety [7], whereas fat exerts the weakest effect intake [8], although they are able to induce ileal brake [9]. There is some controversy regarding fiber effectiveness [10,11].

Polyphenols, a large group of molecules found in a myriad of plant sources, have also been postulated as possible modulators of food intake. Panickar summarized that some polyphenols (resveratrol, apigenin) could act on neuropeptides involved in food intake control, such as Neuropeptidy Y (NPY), Agouti-related protein (AgRP), Pro-opiomelanocortin (POMC) and Cocaine- and amphetamine-regulated transcript (CART) [12]. However, information about the effect of polyphenols on peripheral tissues is scarce, and there is no clear consensus regarding the effects of the most studied compounds, isoflavones [13]. We have previously reported that flavanols, a type of polyphenol found in abundance in grape seed proanthocyanidin extract (GSPE), have a modulatory effect on food intake. We described a dose that inhibited food intake and stimulated energy expenditure; this dose inhibited body weight gain by 60% after two eight-day periods of treatment [14]. Previously, we have shown that GSPE produces a lipolytic effect, which contributed to the effects on body weight in this study [12,15,16]. We ruled out the possibility that proanthocyanidins were playing an important role in inhibiting the gastrointestinal digestion process [14]. Remarkably, the animals ingested less food, which could be due to the changes induced by the extract on enteroendocrine signals (increased active GLP-1 [17] and decreased acylated ghrelin [18]) and/or by other signals that limit their need to feeding [17]. Bao et al, working with GSPE in diabetic rats and at doses similar to ours, also found an inhibition in food intake after chronic administration [19]. However, the limited human studies conducted to date have yielded controversial results. Some studies reported no effect by flavanols on food intake [20,21]. Törrönen et al. found increases in Glucagon-like peptide-1 (GLP-1) after the administration of a berry purée containing proanthocyanidins, although they reported no effects on food intake [22]. For an improved understanding of the effects of GSPE, it is therefore important to describe more extensively its mechanisms of action and the factors that influence it.

In the present study, we fine-tune the mechanisms of the effects of GSPE on food intake in rats. We describe how reproducibility of this effect is limited by stress, synchronicity and dose. We also prove the importance of GLP-1 in the GSPE effects on food intake and its main target tissues.

## 2. Materials and Methods

### 2.1. Materials

The proanthocyanidin-enriched GSPE was obtained from *Les Dérivés Résiniques et Terpéniques* (Dax, France; Batch number: 124029). According to the manufacturer, the extract contains monomeric (21.3%), dimeric (17.4%), trimeric (16.3%), tetrameric (13.3%) and oligomeric (5–13 units; 31.7%) proanthocyanidins. GSPE were characterized in more detail by reverse-phase chromatographic analyses by our research group [23]. Briefly, the main compounds (up to tetramers) found in the GSPE lot used in this study were gallic acid (31 mg/g), (epi)catechin (214 mg/g), epicatechin gallate (21 mg/g), procyanidin dimers (168 mg/g), procyanidin dimer gallate (9 mg/g) and trimers (5 mg/g). The phenolic content of the extracts was quantified using the Folin method [24] as 845.5 ± 10.5 mg/g GSPE. Gallic acid, exendin 9-39 (Ex9) and 70 kDa fluorescein isothiocyanate (FITC)-dextran were obtained from Sigma (St. Louis, MO, USA). GLP-1 7-36 amide was obtained from PolyPeptide (Limhamn, Sweden).

### 2.2. Animals

Female Wistar rats (Harlan, Barcelona, Spain), weighing 180–200 g upon arrival, were used for the food intake studies. A group of adult male Wistar rats (Harlan, Barcelona, Spain), weighing 450–500 g, were used for food intake studies, as a model for glucose-impaired tolerance [25]. The male groups were studied at the facilities of the Technological Center of Nutrition and Health (www.ctns.cat). All the procedures were approved by the Experimental Animal Ethics Committee of the Universitat Rovira I Virgili (code: 0152S/4655/2015).

On arrival, the animals were single housed at 22 °C under a 12 h light/dark cycle (lights on at 8 a.m.) with access to standard chow pellets (Teklad Global Diets #2014, Harlan, Barcelona, Spain) and tap water ad libitum for a one-week adaptation period. Following this adaptation period, the animal sets for the food intake studies were deprived of food every day for 4 h before between 16:00 and 20:00, and chow intake measurements were taken on the 4 days prior to each experiment to habituate the subjects to the experimental schedule. One cross-over experiment per week was performed for all of the food intake studies.

### 2.3. Food Intake Experiments

**Acute treatments.** To assess the effects of different acute doses of proanthoacyanidins on food intake, trained animals were treated with intragastric GSPE doses (i.g.) 1 h before the dark onset, using tap water as a vehicle, and food intake was measured 12 h after the initiation of the feeding period. Parallel controls were performed by intragastrically administering the vehicle. This experiment was conducted on animals on a standard chow diet and on animals receiving hypercaloric meals as detailed in [17].

**Chronic treatments.** For the chronic treatment assays, animals received two periods of treatment as detailed previously [14]. Briefly, in the first-period treatment, trained animals were divided into 3 groups: a control group treated with a vehicle (tap water) and two GSPE groups (0.5 g GSPE/kg body weight (BW) and 1 g GSPE/kg BW) treated with 423 and 846 mg phenolics/kg of BW, respectively. The treatments were intragastrically (i.g.) administered 1 h before the dark onset for 8 consecutive days, and the chow intake was measured. The animals were then left to resume their standard growth pattern for 30 days before an identical treatment period was repeated. On the ninth day, after fasting from 15:00 to 18:00, the animals were anaesthetised with 70 mg/kg BW i.p. of sodium pentobarbital and were sacrificed by exsanguination of the aortal vein. Tissue samples were immediately frozen in liquid nitrogen and then stored at −80 °C.

**Gastrointestinal motility.** To assess the effects of GSPE on gastrointestinal motility, rats were treated 1 h before light offset with tap water or 0.5 g GSPE/Kg BW using 2.5 mg of 70 kDa FITC-dextran as a vehicle. Chow was returned to the cages at light offset and the rats were sacrificed 20 min after by aortal exsanguination under sodium pentobarbital anesthesia. After the excision of the stomach and caecum, the colon was divided into 2 equal parts, and the small intestine was divided into 10 equal parts. The contents of the stomach, caecum and each intestinal segment were washed in 5 mL PBS and the FITC-dextran of each segment were quantified in clean supernatants after a 5 min centrifugation at 12,000 rpm. To determine the motility of FITC-dextran, the gastrointestinal segments were indexed from 1 (stomach) to 14 (intestine, caecum and colon) and the FITC-dextran content was multiplied by its corresponding index to obtain a mean geometric center of motility [26,27,28].

**Antagonism study.** To prevent any stress from interfering with food intake, prior to the experiments with the GLP-1 receptor antagonist Ex9, the rats were specially trained by being handled daily for 10 s at 30 and 15 min before light offset for 2 weeks. A blank food intake measure was taken 3 days prior to an experiment. For the 2 days prior to any experiment, the rats were given a sham dose at 30 and 15 min before light offset, and blank measurements of chow intake were taken. Experiments were performed only if normal food intake was detected after the sham dosage. A crossover experiment was performed once a week. Rats were treated i.p. with saline or Ex9 30 min before light offset and thereafter with intragastric water, gallic acid or GSPE 15 min before light offset. Chow intake was subsequently measured after it was reintroduced [29,30,31]. The Ex9 dose was studied previously to rule out any effect on food intake and to confirm its antagonistic effect on i.p. injected GLP-1.

### 2.4. Plasma and Tissue Quantification

Plasma leptin was assayed in duplicate using a commercial ELISA kit (Millipore, St. Charles, MO, USA). Total RNA was extracted using Trizol (Invitrogen, Carlsbad, CA, USA) and trichloromethane-ethanol (Panreac, Barcelona, Spain), and purified using an RNA extraction kit (Qiagen, Hilden, Germany). Complementary DNA was obtained using the High Capacity cDNA Reverse Transcription Kit (Applied Biosystems, Madrid, Spain), and the quantitative reverse transcriptase-polymerase chain reaction (qRT-PCR) amplification was performed using TaqMan Universal PCR Master Mix and the respective specific TaqMan probes (Applied Biosystems, Madrid, Spain). The relative expression of each mRNA was calculated against the control group using the 2^−ΔΔ*C*t^ method, with actin and cyclophilin as a reference.

### 2.5. Statistical Analysis

Single cosinor analysis and Rayleigh *z*-tests were used to assess the synchronicity of food intake as described previously [32,33,34]. The effects of GSPE on gastrointestinal motility, gene expression, gallic acid effects on food intake and the antagonism study were assessed by Student’s *t*-test. The dose-response effects of GSPE on food intake and the stressing effects of Oral Glucose Tolerance Test (OGTT) on food intake were assessed by one-way ANOVA and appropriate post hoc tests. The role of the hypothalamic gene expression of neuropeptides in food intake, glucagon and GLP-1 receptor gene expression in the hypothalamus after the chronic treatment was assessed by multivariate linear regression analysis for each treatment group. The statistical analyses were performed with IBM SPSS Statistics 22 (IBM Corp., Armonk, NY, USA). *p*-values < 0.05 were considered significant in all cases. The data are represented as mean ± SEM.

## 3. Results

### 3.1. The Importance of Stable Eating Patterns for Determining the Effects of Procyanthocyanidins on Food Intake

**Figure 1:**
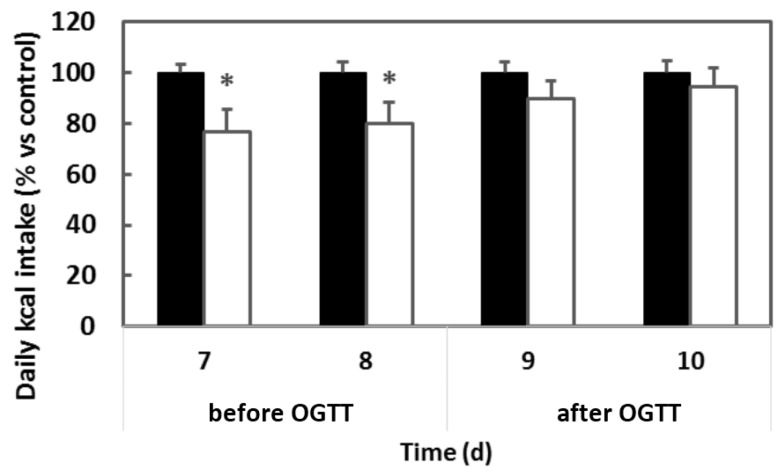
Effect of stressing treatment on food intake measurement. Wistar rats received grape seed proanthocyanidins (GSPE) treatment (0.5 g/kg body weight (BW)) (**white** columns) on the days shown (*x*-axis). Control animals are indicated as **black** columns. Food intake was measured 20 h after the start of the dark-phase start. On day eight after GSPE treatment initiation, rats were overnight fasted and exposed to an Oragl Glucse Tolerance Test (OGTT) the next morning. The results are shown as % vs. control group. *n* = 6 per group; * *p* < 0.05 vs. control group by Bonferroni test after ANOVA.

We first observed that stress removes the inhibitory effect of GSPE on food intake. Figure 1 shows that, in aged males, GSPE had a clear inhibitory effect on food intake that could be observed daily during the first eight days of treatment, but that this effect was lost on the ninth day when animals were subjected to eight hours of overnight fasting and followed by an OGTT, a procedure that involves several stressing situations as overnight fast, forced oral glucose administration and blood sampling from tail tips.

Another key point in the feeding physiology is the existence of feeding patterns. Food intake has been reported as having a periodicity of close to 3.5 days for male Wistar rats [33]. In the case of female rats, food intake shows a periodicity consistent with the four-day estrous cycle [35]. Single cosinor analysis showed that the food intake of both male and female rats follows a four-day cycle, with a greater minimum to maximum amplitude among females, which could reflect the influence of the estrous cycle (males: 12% ± 1%, females: 25% ± 4%; relative to the mean food intake in the period). We performed Rayleigh a test to check the effects of GSPE on the synchronicity of the food intake patterns [34]. Table 1 shows that the control animals that lived together for more than two weeks are perfectly synchronized in the first treatment study (F1) (critical *r* for *p* < 0.05 = 0.69). The GSPE-treated rats did not adjust to the same extent as the control animals, suggesting a change in the feeding pattern of these animals. This effect was even stronger for the highest dose (1000 mg GSPE/kg BW). We found a similar change in the synchronization when we reproduced food intake measurements in the same control group (indicated as C (F2) in Table 1), but this time starting treatment by pairs on sequential days without enough time for synchronization between them.

### 3.2. The Minimal Dose of GSPE Required to Limit Food Intake

**Figure 2:**
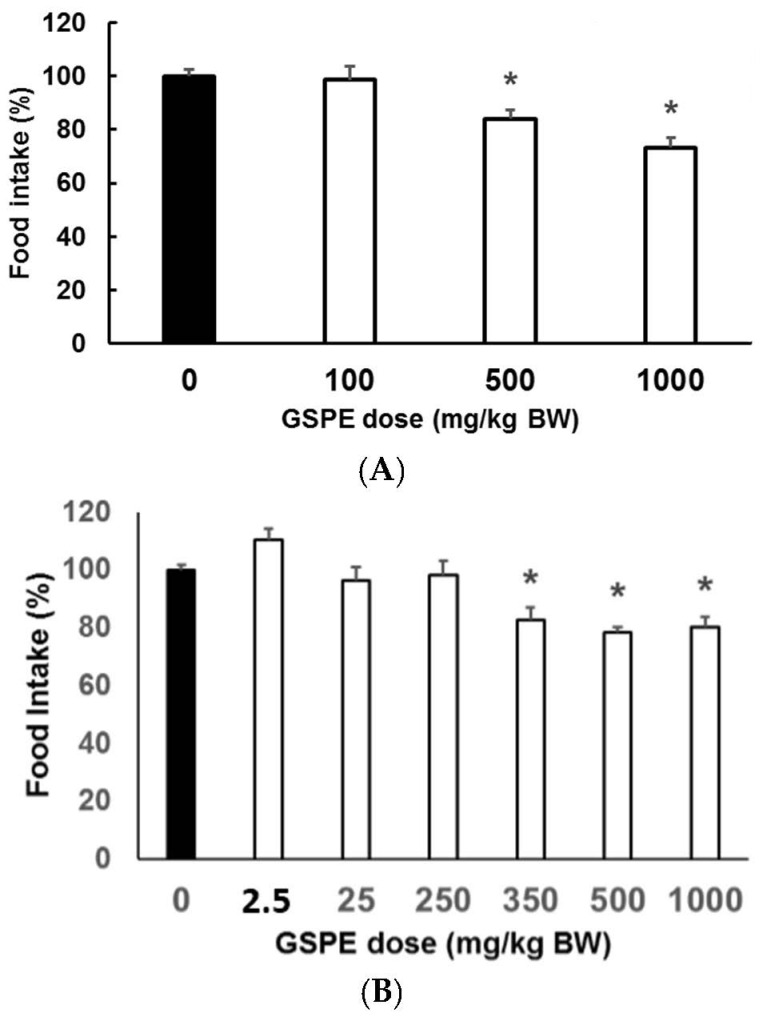
Range of effective doses at which grape seed proanthocyanidin extract (GSPE) inhibits food intake. Wistar rats received one of the various GSPE doses (**white** columns). Control animals are indicated as **black** columns. Food intake was measured twelve hours after the initiation of food intake period. (**A**) rats on a highly palatable diet; and (**B**) rats fed with standard chow. Results are shown as % vs. control group. *n* = 6 per group; * *p* < 0.05 vs. control group by Dunett’s test after ANOVA.

To identify the minimal dose needed to obtain the inhibitory effect of GSPE on food intake, we assayed several GSPE doses (100, 500 and 1000 mg GSPE/kg BW) using synchronized females on a highly palatable diet. Figure 2a shows that a minimal dose of 500 mg GSPE/kg BW is needed to produce a statistically significant inhibition of food intake in animals fed a highly palatable diet. Since a highly palatable diet broke the normal food pattern of rats fed on standard chow, we also assayed different doses in rats fed on standard chow. Figure 2b shows a statistically significant inhibition of food intake in rats fed on standard chow after the minimum dose of 350 mg GSPE/kg BW.

Importantly, the upper effective dose assayed had collateral effect of increasing the caecum size (g of tissue: 1.91 ± 0.12 GSPE group vs. 1.47 ± 0.1 control group; g of content: 8.22 ± 0.39 GSPE group vs. 4.3 ± 0.3 control group).

### 3.3. GLP-1 Plays a Major Role in Mediating GSPE Inhibitory Effects on Food Intake

**Figure 3:**
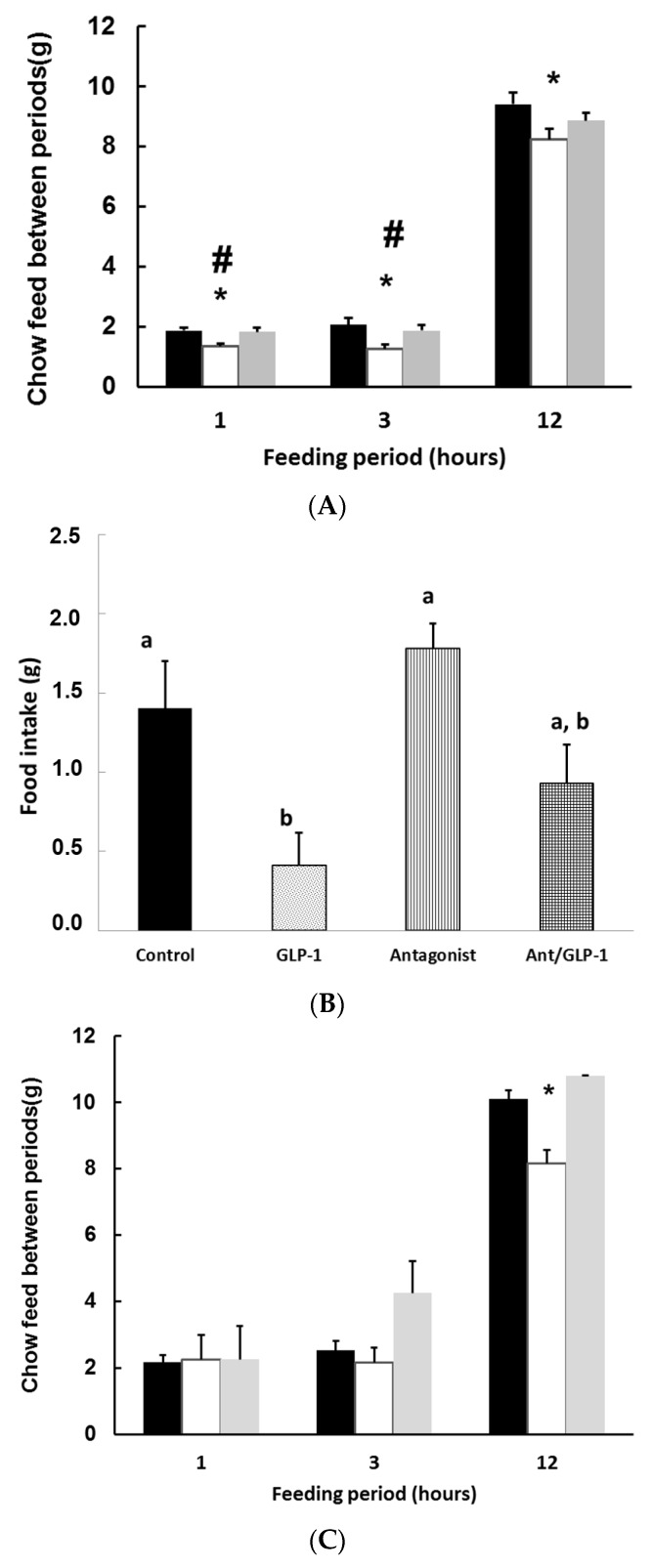

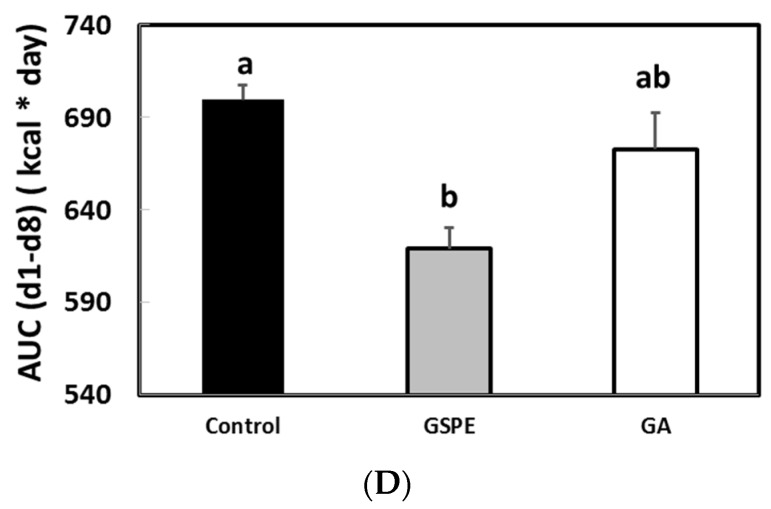
Antagonism of Exendin (9-39) on the effects of grape seed proanthocyanidin extract (GSPE) and gallic acid on food intake. (**A**,**B**) Wistar rats received one acute oral GSPE or i.p. Glucagon-like peptide-1 (GLP-1) treatment, with or without Exendin (9-39) prior to the start of the food intake period. Food intake was measured at the time intervals indicated, counted from the initiation of the dark phase. The **black** column refers to the control group: (**A**) the **white** column refers to GSPE treatment of 0.5 g/kg body weight (BW); the **grey** column indicates those animals that previously received treatment with Exendin (9-39); (**B**) food intake measurements were at 3 h after the food initiation period. *n* = 14 per group. # *p* < 0.05 vs. antagonist. * *p* < 0.05 vs. control group. Different superscripts indicate statistical differences between the treatments by *t*-test; (**C**,**D**) relate to the effects of gallic acid on food intake: (**C**) animals received one dose of treatment. The **white** column refers to gallic acid treatment; the **grey** column indicated those animals that previously received treatment with Exendin (9-39); (**D**) refers to animals treated for eight days with the indicated treatments. *n* = 9 per group * *p* < 0.05 vs. control group by *t*-test. Different superscripts (lowercase in panels B and D) indicate statistical differences between the treatments.

Given that GSPE was effective in increasing active GLP-1 levels [36] at doses inhibiting food intake [17], we checked the role of GLP-1 in this effect on food intake. Figure 3a shows that the short-term effects of GSPE on food intake were antagonized by the GLP-1 receptor (GLP-1-R) antagonist Ex9 [29], as was the case with i.p. GLP-1 treated rats (Figure 3b).

Since we were working with an extract, we were interested in identifying the main ingredient responsible for this effect on food intake. We checked whether gallic acid (for which a food intake inhibitory effect has also been defined [37]), could play a role in the effects of GSPE on food intake. Figure 3c shows that gallic acid administered in the equivalent dose as in the GSPE treatment had an effect on food intake 12 h after the initiation of the feeding period.

Furthermore, the effects of equivalent doses of gallic acid and GSPE on food intake inhibition were different after a chronic treatment. We found that gallic acid exhibited lower effects than the GSPE extract, as shown in Figure 3d.

### 3.4. Key Organs for the Inhibitory Effect of GSPE on Food Intake 

In order to identify where GSPE acts to modulate food intake, we assayed the changes in the main organs involved in controlling food intake. To do this, we analyzed gastrointestinal derived signaling, peripheral signals and the central nervous system.

**Figure 4:**
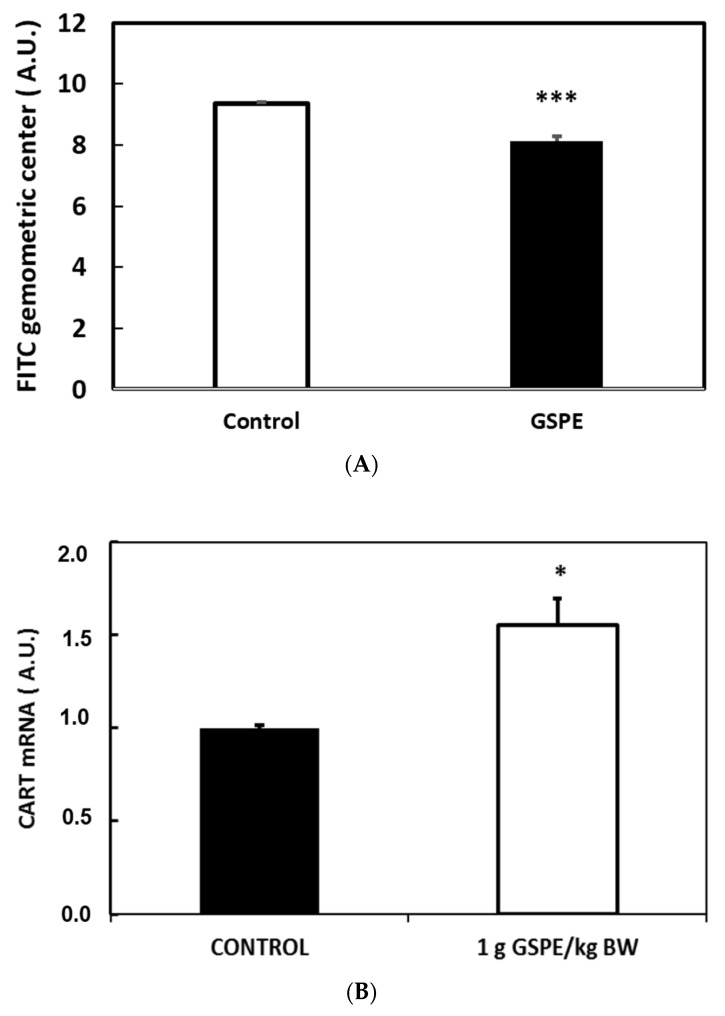
Grape seed proanthocyanidin extract (GSPE) effect on physiological parameters. Wistar rats that received GSPE treatments are indicated as **white** columns. Control animals are indicated as **black** columns. (**A**) rats received a chronic treatment with 0.5 g GSPE /kg body weight (BW). Fluorescein isothiocyanate (FITC) was orally forced and FITC in different gastrointestinal localizations was measured after death; (**B**) rats received an acute treatment of 1 g GSPE/kg BW. After death, hypothalamic CART expression was measured. *n* = 6 per group; * *p* < 0.05; *** *p* < 0.001 vs. control group by Student’s *t*-test.

Figure 4a shows a higher FITC content in the upper gastrointestinal tract, indicating lower gastrointestinal motility as a result of GSPE treatment. To analyze peripheral signaling, we assayed changes to leptinaemia after eight days of GSPE treatment. There were no differences in leptin levels between the groups (0.5 dose: 13.2 ± 5.04; control: 17.78 ± 1.82 (ng/mL)).

To evaluate effects on the central nervous system, we analyzed changes in the expression levels of the main hypothalamic neuropeptides. We found that in rats who fasted, after an acute dose of 1 g GSPE/kg BW, which increased GLP-1 plasma levels, CART mRNA expression, increased slightly, with no changes in the other assayed neuropeptides (Figure 4b). After chronic treatments, with doses limiting food intake, we found no clear effects on the hypothalamic mRNA levels of GLP-1, GLP-1-R, POMC/CART and NPY/AgRP, but there was a very strong statistical correlation between them. In order to evaluate the effect of GSPE on hypothalamic gene expression, we analyzed the multivariate linear regression between mRNA levels and food intake in these animals. Table 2a shows that 79% of food intake in the control animals was explained statistically by hypothalamic POMC expression (first row). GSPE treatments changed this relation, suggesting an effect on the hypothalamic control of food intake. This effect was more evident in the dose of 1 g GSPE/kg BW, where a statistically significant 79% of food intake could be explained by the hypothalamic GLP-1 expression.

Sisley et al. argued that neuronal GLP-1Rs mediate the anorectic effects of liraglutide, a long-lasting GLP-1 agonist [38]. As shown in Table 2b, we checked if GSPE modulated the relations between the expressions of this receptor and the food intake control neuropeptides (AgRP/POMC/NPY/CART). mRNA expression of GLP-1 receptor fits perfectly (100%) with the expression of CART and NPY in control rats. GSPE treatments also abolished the relationship between NPY and GLP-1-R. Likewise, in the control group the correlation between AgRP and POMC accounts for 94% of the changes in the GLP-1 receptor (*p* = 0.059), in a relationship that is also modified by GSPE. Despite the possibility that 86% of the changes in the GLP-1 receptor after the 0.5 GSPE dose were due to POMC mRNA (Table S1 included as supplementary material), there was a lack of correlation between GLP-1 mRNA levels and the mRNA of the other neuropeptides.

## 4. Discussion

We initially defined the inhibitory effect of GSPE on food intake in female, male, young, aged, normofagic and hyperfagic animals after an acute treatment [17]. Afterwards, we showed that a specific dose is needed to ensure a significant chronic effect on body weight control [14]. We now describe in deeper detail several aspects that should be taken into account if a GSPE treatment is to be effective.

According to our previous results, to obtain an inhibition on food intake, GSPE needs to be administered prior to or simultaneously with food intake [14]. Here, we first showed that to obtain an inhibition on food intake, animals have to be maintained as far as possible in calm, stress-free conditions. Abbott et al. lost the anorectic effect of Peptide YY (PYY) and GLP-1 administration simply by moving the rats from their original cage to a new cage [39]. In our study, we lost the effectiveness of our treatment through overnight fasting, the forced administration of glucose and the tail blood sampling required to carry out for an OGTT.

Regarding the optimal dose, we initially proved that the inhibition on food intake could be obtained by the GSPE dose, which caused an increase in GLP-1 levels, 1 g GSPE/kg BW; we then proved that GSPE at a dose of 500 mg of GSPE extract/kg of body weight is the optimal dose [17]. However, since several studies have failed to find any effect on food intake at lower doses, we considered it necessary to determine the range of doses that could inhibit food intake. To address this point, it should be recalled that the rats’ feeding behavior was perfectly adjusted under a normal food pattern with standard chow, but that this was disrupted under a highly palatable diet [40]; consequently, it was essential to evaluate the effectiveness under both situations. Working with healthy female rats after fifteen days of adaptation to synchronize their estrus cycles before studying food intake [41], we again found that 500 mg/kg BW dose is the minimal amount of GSPE required under a stimulated food intake situation. However, we also found that this dose could be reduced to 350 mg/kg BW for the animals fed standard chow. Bao and et al. [19], working with Diabetes Mellitus animals fed with standard chow, also showed a similar effect working with a similar extract. They also showed the need for a minimal dose, since, in their study, a dose of 125 mg GSPE/kg BW did not reduce food intake, while a dose of 250 produced a 15% food intake inhibition, which increased to 25% with a dose of 500 mg/kg BW. Furthermore, we identified certain undesirable effects when 1000 mg GSPE/kg BW was administered chronically, which leads us to the conclusion that an upper effective limit also needs to be established. This dose did not maintain food intake inhibition after a second treatment period, and it did not increase the animals’ energy expenditure [14], probably as a result of the exaggerated effects during the first treatment period. We have showed some differences in gastrointestinal physiology and in the mechanism of action between the assayed doses that could explain the different levels of effectiveness in terms of metabolic parameters. This dose or higher ones have been shown to be nontoxic [42,43]; however, studies on toxicity did not measure food intake after chronic treatment. Consequently, our results lead us to the conclusion that GSPE treatment requires a range of optimal doses to be effective. This could also explain why so few studies have demonstrated a lack of the effects of flavanols on human food intake, since most of studies have used doses lower than suggested for humans in the present study (100 mg GSPE/ Kg BW according to Body Surface Area methodology [44]) [20,21,22].

Given that food intake control involves several key organs in the body, we tried to identify the main target tissue for GSPE in terms of its effect on food intake. At the gastrointestinal level, we have shown that GSPE modifies several enteroendocrine secretions and delays gastric emptying [17]. We also have shown a delay in intestinal motility after the final dose of an eight-day treatment with 0.5 g/kg BW in animals that were awake, thus reproducing the previously shown effect in anesthetized animals after a single 1 g/kg BW dose with GSPE [17]. This delaying effect is a key mechanism in the short-term limitation of food intake caused by GLP-1 [45,46]. We previously proved that GSPE increased active GLP-1 [36]. In the present study, we have shown that antagonism of the GLP-1-Receptor removes the GSPE effect on food intake, thus suggesting that the increase in GLP-1 is a key mediator regarding the effect of GSPE on food intake. Similar results were obtained by Sisley et al. working with liraglutide (a long-lasting GLP-1 agonist) [38]. From our data, we also identified for the first time that GSPE affects the standard pattern of the feeding profile in male rats. Ribas-Latre et al. showed that acute similar doses of GSPE could have chronobiological properties, depending on the time of administration [47]. In our studies, the time of administration is a constant parameter between the groups. Since GLP-1 levels have also been shown to be sensitive to circadian rhythms [48], it could be possible that the effects of GSPE on feeding patterns are related to the effects of GSPE on GLP-1.

If GLP-1 produced at gastrointestinal level is one of the signaling molecules that mediates the effect of GSPE on food intake, hypothalamic neuronal centers need to be sensitive to this treatment. GLP-1 directly activates POMC/CART neurons and, via GABAergic transmission, indirectly inhibits the neuropeptide Y/agouti-related peptide (NPY/AgRP) neurons, which collectively results in signals that reduce food intake [49]. A previous review showed that there is a lack of information regarding the effects of flavanols on food intake and the neuropeptides controlling food intake [12]. We showed that chronic GSPE treatment for 12 weeks, at a dose without an effect on food intake, concomitant with a cafeteria diet, increased the expression of hypothalamic GLP-1 and decreased GLP-1-receptors [50]. In the present study, we have shown that there is a different pattern between the relations defined for the control animals regarding food intake and the hypothalamic peptides resulting from GSPE treatment. This effect differs depending on the dose. By comparing the relation between hypothalamic GLP-1-Receptor expression and hypothalamic peptides, we also found that the control group had a different pattern from the GSPE treated animals, all of which suggests that GSPE affects the hypothalamic centers involved in controlling the food intake.

To determine whether peripheral signals that indicate the status of the body’s energy storage have a role in food intake control, we analyzed leptinemia and found no changes due to GSPE treatments. Chronic long-term treatment with doses without properties on food intake have proven effective in limiting the increase in leptinemia related to a high-fat diet in hamsters [51,52], but corrective GSPE doses for shorter periods (30 days) did not revert the higher leptinemia [51]. Thus, the different studies, working with different doses, show inconsistent results that prevent clear conclusions from being drawn regarding the effect of GSPE on leptin production.

Another aspect that needs to be addressed is identifying the compound/s in the extract that exert the inhibitory effect on food intake. Through a comparative study between GSPE and Cocoa extract (Cocoanox), we had previously ruled out epicatechin, catechin and their oligomeric and polymeric forms as the main candidates because Cocoa extract, which is rich in these forms, was less effective at inhibiting food intake than GSPE [17]. In contrast to Cocoanox, GSPE contains gallic acid and gallated flavanols. Our results showed that gallic acid, for which effects on food intake have previously been defined [37], could therefore also be involved in the effect of GSPE limiting food intake. In fact, the GLP-1 antagonist Ex9 also inhibits the effects of gallic acid limiting food intake, suggesting that gallic acid needs the participation of GLP-1. In support of this, we have also proved that gallic acid inhibits intestinal Dipeptidyl peptidase-4 (DPP4) activity, thus favoring an increase in GLP-1 intestinal production [36]. However, gallic acid only showed clear effects after several hours of treatment, an aspect that contrasts with the high bioavailability of gallic acid, which has been identified at around 60–90 min after ingestion in rats and humans [53,54]. Therefore, gallated flavanols might be responsible for the short-term effects of GSPE at inhibiting food intake. To support this hypothesis, it should be recalled that bitter compounds directly interact with gastrointestinal smooth muscles to inhibit gastrointestinal motility, thus producing a feeling of fullness and diminishing food intake [55,56,57]. Since the bitter-sensing proprieties of flavanols increase in the presence of a galloyl moiety in the 3rd position of the C-ring [58], the gallated flavanols in GSPE could play a main role in this short-term inhibitory effects. Proving this hypothesis is severely limited by the difficulties faced in obtaining pure gallated forms for assays in animals.

## 5. Conclusions

In conclusion, GSPE must be used at an optimal dose under non-stressful conditions with an appropriately defined pattern of administration to act as limiting agent on food intake. The bioactive components of the extract act by modifying the GLP-1 signaling, which acts on the central areas that control food intake. Some more work is needed to adjust their use on humans.

## Figures and Tables

**Table 1:** Rayleigh *z*-test to measure effects on feeding pattern synchronicity.

Treatment	*n*	Vector Size (*r*)
C (F1)	6	0.72 *
500 mg GSPE/kg BW	6	0.56
1000 mg GSPE/kg BW	6	0.34
C (F2)	6	0.68

F1, first treatment study; F2, second treatment study; GSPE, grape seed proanthocyanidin extract; BW, body weight; * *p* < 0.05 vs. control (C) group.

**Table 2:** Multivariate linear regression after chronic grape seed proanthocyanidin extract (GSPE) treatment.

(**a**)							
**Treatment**	**a**	**X_1_**	**b**	**X_2_**	**c**	***p***	***R*^2^**
Control	55.54	POMC			343.6	0.044 *	0.79
0.5 GSPE	9.08	POMC			373.9	0.426	0.33
1 GSPE	2.67	POMC	2.24	CART	435.5	0.835	0.17
Control	29.85	GLP-1h			444.0	0.345	0.22
0.5 GSPE	−10.22	GLP-1h			423.6	0.899	0.01
1 GSPE	21.18	GLP-1h			425.7	0.045 *	0.79
(**b**)							
**Treatment**	**a**	**X_1_**	**b**	**X_2_**	**d**	***p***	***R*^2^**
Control	0.37	CART	−0.25	NPY	0.37	0.000 *	0.99
0.5 GSPE	2.94	CART	1.06	NPY	−10.55	0.772	0.41
1 GSPE	0.30	CART	0.56	NPY	−0.46	0.389	0.85
Control	0.70	POMC	−0.87	AgRP	0.49	0.059	0.94
0.5 GSPE	0.36	POMC	-	-	0.40	0.022 *	0.86
1 GSPE	−0.01	POMC	1.63	AgRP	−0.36	0.525	0.72

Wistar rats treated for eight days with 0.5 g GSPE/kg body weight (BW) or 1 g/kg were killed under fasting conditions. mRNA levels of POMC, NPY, AgRP, CART, GLP-1 and GLP-1-R were evaluated in the hypothalamus to determine their fit with the multivariate linear regression of Y = aX_1_ + bX_2_ + c. (**a**) Y: food intake from these animals; (**b**) Y: GLP-1 Receptor mRNA in hypothalamus *p*-value < 0.05 indicates a statistically significant regression. *R*^2^ identifies the degree of fit between related parameters.

## Supplementary Materials

The following are available online at http://www.mdpi.com/2072-6643/8/10/652/s1, Table S1: Y: Multivariate linear regression. Y: GLP-1 mRNA in hypothalamus.
