# Supplementary material for: Defining Conditions for Optimal Inhibition of Food Intake in Rats by a Grape-Seed Derived Proanthocyanidin Extract
Source: Nutrients. 2016 Oct 20;8(10):652. doi: 10.3390/nu8100652 (PMC5084038; doi:10.3390/nu8100652)
Supplement: Supplementary file 1 [file nutrients-08-00652-s001.docx]

Supplementary Materials: Defining Conditions for Optimal Inhibition of Food Intake in Rats by a Grape-Seed Derived Proanthocyanidin Extract

Joan Serrano, Àngela Casanova-Martí, Mayte Blay, Ximena Terra, Anna Ardévol
and Montserrat Pinent

**Table S1.** Y: Multivariate linear regression. Y: GLP-1 mRNA in hypothalamus.

| **Treatment** | **a** | **X_1_** | **b** | **X_2_** | **d** | ***p*** | ***R*^2^** |
| --- | --- | --- | --- | --- | --- | --- | --- |
| Control | 0.32 | CART | −2.02 | NPY | 2.31 | 0.777 | 0.15 |
| 0.5 GSPE | 0.06 | CART | 1.93 | NPY | −1.68 | 0.555 | 0.69 |
| 1 GSPE | 0.09 | CART | 0.02 | NPY | 0.79 | 0.909 | 0.09 |
| Control | 0.38 | POMC | 1.44 | AgRP | 0.38 | 0.667 | 0.33 |
| 0.5 GSPE | 1.04 | POMC | 0.047 | AgRP | 1.04 | 0.949 | 0.05 |
| 1 GSPE | 0.80 | POMC | 0.201 | AgRP | 0.80 | 0.96 | 0.04 |

GSPE, grape seed proanthocyanidins; NPY, Neuropeptidy Y (NPY); AgRP, Agouti-related protein; POMC, Pro-opiomelanocortin; CART, Cocaine- and amphetamine-regulated transcript; GLP-1, Glucagon-like peptide-1.
